# Epidemiological characteristics and seasonal dynamics of six respiratory pathogens in children: a 2-year retrospective cross-sectional study in Chengdu, China

**DOI:** 10.1186/s12887-026-07063-3

**Published:** 2026-06-03

**Authors:** Hongjia Chen, Yinghong Fan, Yijie Huang

**Affiliations:** Chengdu Women’s and Children’s Central Hospital, School of Medicine, University of Electronic Science and Technology of China, 1617 Riyue Avenue, Qingyang District, Chengdu, Sichuan Province China

**Keywords:** Pediatric acute respiratory infection, Multiplex PCR, Rhinovirus, Respiratory syncytial virus, *Mycoplasma pneumoniae*, Post-COVID-19

## Abstract

**Background:**

Adenovirus (AdV), influenza A virus (FluA), influenza B virus (FluB), respiratory syncytial virus (RSV), rhinovirus (RV), and *Mycoplasma pneumoniae* (MP) are the primary pathogens causing acute respiratory infections in children. However, comprehensive and multifactorial analyses of their epidemiological features, such as seasonality and age specificity, remain limited.

**Methods:**

We performed a retrospective analysis of 68,982 children (aged 0–18 years) with acute respiratory infections who underwent multiplex PCR testing at Chengdu Women and Children’s Central Hospital between March 1, 2023, and February 28, 2025. Single-pathogen positivity rates were compared using Pearson’s chi-square test. Multivariate logistic regression was used to evaluate the associations between age group, season, sex, and care setting and pathogen detection, reporting adjusted odds ratios (ORs) and 95% confidence intervals (CIs) with adolescence, autumn, female sex, and inpatient status as reference categories. Co-infection patterns were analyzed by constructing a network based on pairwise co-detection frequencies, calculating node degrees and edge weights to identify predominant mixed-infection combinations.

**Results:**

Overall PCR positivity was 53.1% (36,677/68,982). The positivity rate increased from 38.8% in infants to 63.5% in school-aged children (χ² = 2276.7; *P* < 0.001). Among all pathogens, RV remained common across all age groups, with an overall positivity rate of 18.1%. Compared to adolescents, infants had significantly higher odds of RSV detection (OR = 7.82; 95% CI 5.53–11.04); school-aged children had higher odds of MP detection (OR = 2.82; 95% CI 2.39–3.32). Males exhibited slightly higher overall positivity than females (53.6% vs. 52.6%; χ² = 7.41; *P* = 0.007). Positivity was higher in winter and autumn than in summer (58.1% vs. 46.6%; χ² = 628.6; *P* < 0.001). Outpatients had higher positivity rates than inpatients (63.2% vs. 51.1%; χ² = 586.7; *P* < 0.001). Seasonal analysis showed that AdV peaked in summer (OR = 1.46; 95% CI 1.38–1.55); RV peaked in autumn and spring (summer OR = 0.62; 95% CI 0.59–0.65); MP peaked in autumn; and RSV and influenza B virus peaked in winter (RSV OR = 2.82; 95% CI 2.63–3.03; FluB OR = 4.70; 95% CI 3.94–5.60). Co-infection analysis revealed that the most common co-infection pattern was RV + MP, with 1,009 cases identified in hospitalized patients.

**Conclusions:**

Age, season, and care setting significantly affect pediatric respiratory pathogen detection. These findings may inform targeted public health surveillance and respiratory infection prevention strategies.

**Supplementary Information:**

The online version contains supplementary material available at 10.1186/s12887-026-07063-3.

## Background

Acute respiratory infections (ARIs) remain a leading cause of paediatric morbidity and mortality worldwide. In 2019, pneumonia alone resulted in approximately 740,180 deaths among children under five, accounting for 14% of all deaths in this age group [1]. The incidence of upper respiratory tract infections reached 162484.8 per 100,000 population in 2021, with the highest burden in children under two years of age [2]. Excluding COVID‑19, lower respiratory tract infections were the fifth leading cause of global death in 2021, and respiratory syncytial virus (RSV) accounted for approximately 3.6 million hospitalizations and over 100,000 deaths annually in children under five [3, 4]. Despite the significant global health burden posed by these infections, comprehensive, large-scale studies examining the epidemiological characteristics of the six most common respiratory pathogens—adenovirus (AdV), influenza A (FluA), influenza B (FluB), RSV, rhinovirus (RV), and *Mycoplasma pneumoniae* (MP)—are limited. These six pathogens were selected for analysis because they represent the primary causative agents of pediatric ARIs, frequently leading to severe clinical outcomes such as bronchiolitis, pneumonia, and asthma exacerbation, which collectively impose a substantial burden on pediatric healthcare resources. Existing research [5–7] is often constrained by small sample sizes, a focus on single pathogens, or the lack of data from outpatient settings, which hinders a broader understanding of their seasonal variations, age-specific prevalence, and co-infection dynamics. This study aims to address these gaps by investigating the epidemiology of these six pathogens in a large cohort of pediatric patients, exploring how age, sex, season, and patient type influence pathogen detection rates. Additionally, we aim to identify key independent determinants of pathogen positivity through multivariable logistic regression and map co-infection patterns to improve targeted surveillance and clinical management strategies.

## Methods

### Study design and subjects

This retrospective cross-sectional study included all children in both inpatient and outpatient settings who presented with acute respiratory infections (ARI) and underwent multiplex PCR testing for six common respiratory pathogens at Chengdu Women’s and Children’s Central Hospital between March 1, 2023, and February 28, 2025. ARI was defined by symptoms such as fever, cough, wheezing, or other upper/lower respiratory manifestations, as per standard clinical practice. Patients with ARI were tested with multiplex PCR according to physician discretion. As the largest specialized pediatric referral center in the Chengdu region, the hospital serves a broad and representative pediatric population from urban and surrounding areas. Patients older than 18 years or those with incomplete clinical or laboratory records were excluded. This study was approved by the Institutional Review Board (IRB) of Chengdu Women’s and Children’s Central Hospital (Approval No.: 2025〔77〕). Data were fully anonymized before analysis.

### Specimen collection and detection methods

Throat swabs were obtained by trained personnel, placed in sterile tubes containing preservation solution, and stored at 2–8 °C for no more than 24 h. Nucleic acids were then automatically extracted using the NATCH 48 system. Amplification was performed on a Gentier 96E real-time PCR instrument with the Sansure Six Respiratory Pathogen Nucleic Acid Detection Kit (Sansure Biotech Co., Ltd., Changsha, China). This multiplex PCR kit is designed to specifically detect the six target pathogens: AdV, FluA, FluB, RSV, RV, and MP, using a single reaction tube for each specimen. Samples showing an S-shaped amplification curve with a Ct value < 40 were considered positive; those without a curve or with Ct ≥ 40 were deemed negative, provided the internal control Ct was < 40—otherwise, the result was invalid. All assays were conducted strictly according to the manufacturer’s protocol, and laboratory staff received standardized training to ensure consistency and accuracy.

### Variable grouping and definitions

Age groups were defined as follows: infants (0–1 year), toddlers (1–3 years), preschoolers (3–6 years), school-aged children (6–12 years), and adolescents (12–18 years).

Seasons were defined as spring (March–May), summer (June–August), autumn (September–November), and winter (December–February).

Visit type was categorized as outpatient versus inpatient.

### Statistical analysis

All statistical analyses were performed using R software (R 4.3.3). Descriptive statistics were used to calculate overall and stratified positivity rates for each pathogen. For between-group comparisons, we employed Pearson’s chi-square test to compare positivity rates across age groups, sex, seasons, and care settings. A two-sided P-value < 0.05 was considered statistically significant. For multivariable logistic regression, age group, sex, season, and care setting were entered into the model simultaneously, with autumn, adolescent, female, and inpatient serving as reference categories. Adjusted odds ratios (ORs) and 95% confidence intervals (CIs) were calculated. For co-infection analysis, frequencies of co-detection combinations were calculated. An UpSet plot was generated using the UpSetR package to visualize the intersection sizes of multiple pathogen co-detections. A network graph was constructed using the R igraph package to analyze pairwise co-infection patterns, with node degree and edge weight calculated to identify major co-infection combinations. Stacked bar charts illustrating the distribution of pathogen detection by sex, year, season, and patient type were created using GraphPad Prism (10.0).

## Results

### Demographic and clinical determinants of pathogen positivity

A total of 68,982 children were screened, and all met the inclusion criteria; no patients were excluded due to incomplete records. Data were fully anonymized before analysis. Among 68,982 pediatric patients, 53.1% (*n* = 36,677) tested positive for at least one respiratory pathogen. Detection rates followed a significant age-dependent gradient (χ² = 2276.7, *P* < 0.001), peaking in school-aged children (63.5%) and reaching the lowest rate in adolescents (37.6%). Males exhibited slightly higher overall positivity than females (53.6% vs. 52.6%, *P* = 0.007). Positivity rates varied significantly by season (χ² = 628.6, *P* < 0.001), with the highest rates in winter (58.1%) and autumn (57.1%) and the lowest in summer (46.6%). Outpatients had a significantly higher positivity rate than inpatients (63.2% vs. 51.1%, *P* < 0.001) (Table 1).

**Table 1:** Positive rate of respiratory pathogen detection by age, gender, season, and visit type

Variables	Total cases (*n* = 68982)	Positive cases (*n* = 36677)	Negative cases (*n* = 32305)	Statistic	*P*
Age group					
Infant, *n*(%)	15,938	6183(38.8)	9755(61.2)	2276.72	< 0.001
Toddler, *n*(%)	15,836	8380(52.9)	7456(47.1)		
Preschooler, *n*(%)	21,046	12,194(57.8)	8842(42.1)		
School-age, *n*(%)	14,855	9429(63.5)	5436(36.5)		
Adolescent, *n*(%)	1307	491(37.6)	816(62.4)		
Gender group					
Male, *n*(%)	38,446	20,619(53.6)	17,827(46.4)	7.41	0.0065
Female, *n*(%)	30,536	16,058(52.6)	14,478(47.4)		
Season group					
Spring, *n*(%)	15,664	7971(50.9)	7693(49.1)	628.64	< 0.001
Summer, *n*(%)	18,225	8493(46.6)	9732(53.4)		
Autumn, *n*(%)	16,373	9342(57.1)	7031(42.9)		
Winter,* n*(%)	18,720	10,871(58.1)	7849(41.9)		
Visit type					
Outpatient, *n*(%)	12,019	7595(63.2)	4424(36.8)	586.71	< 0.001
Inpatient, *n*(%)	56,963	29,082(51.1)	27,881(48.9)		

χ²: Chi-square test

### Pathogen-specific prevalence and clinical setting profiles

**Fig. 1:**
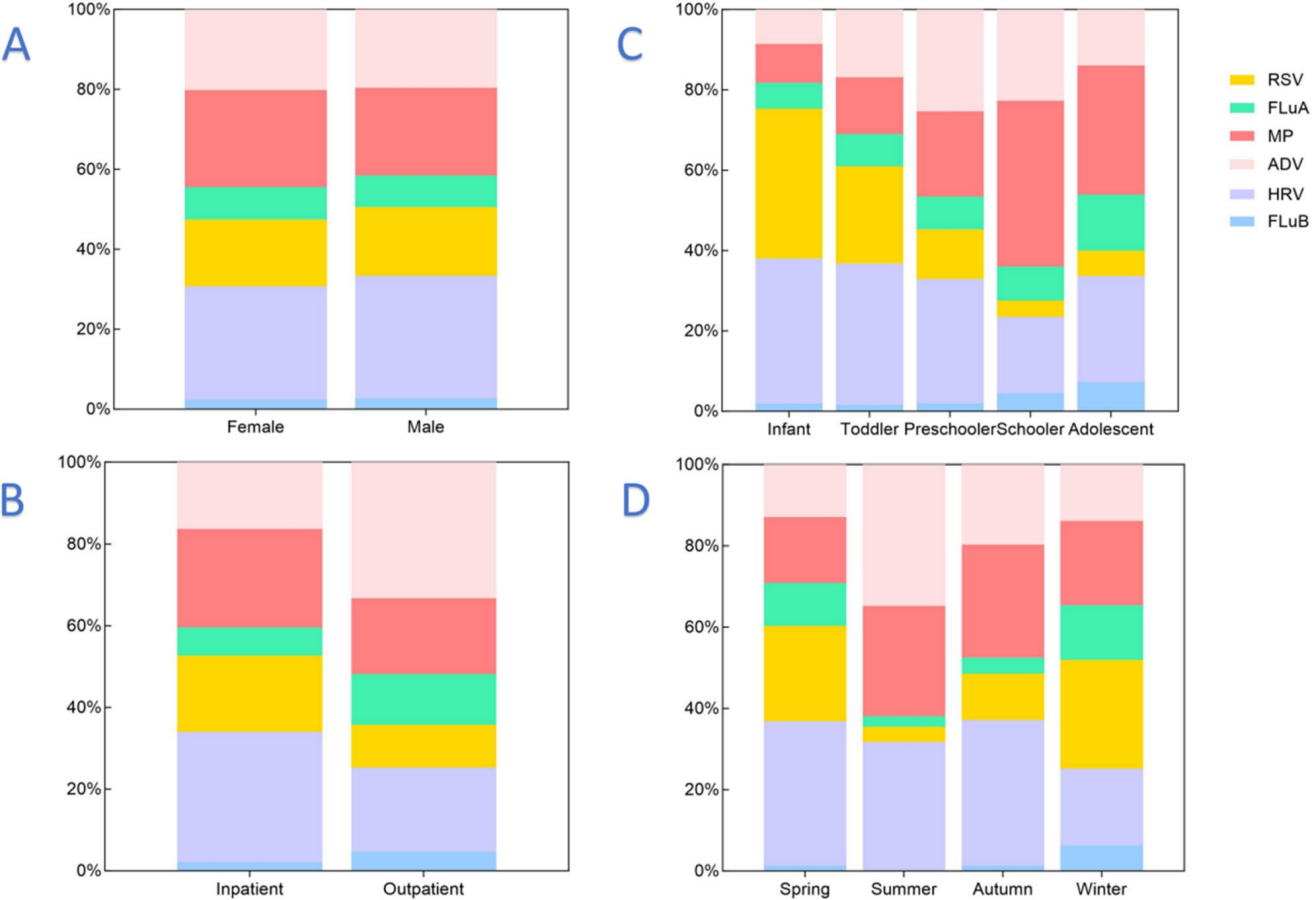
Percentage stacked bar charts of respiratory pathogen composition across different demographic and seasonal subgroups. (**A**: Gender; **B:** Visit Type; **C**: Age Group; **D**: Season)

RV was the most frequently detected pathogen (18.05%), followed by MP (14.02%) and AdV (12.08%). Sex-based differences were observed: RV was more prevalent in males (18.84% vs. 17.07%, *P* < 0.001), while MP was more prevalent in females (14.65% vs. 13.51%, *P* < 0.001). RSV and Flu B also showed higher rates in males (both *P* < 0.05), whereas Flu A and AdV showed no sex differences. Sex-based differences in pathogen profiles are shown in Fig. 1A and detailed in Supplementary Table S1.

The clinical setting strongly differentiated pathogen profiles (Table S2; Fig. 1B). Outpatients had markedly higher detection rates of AdV (24.19% vs. 9.52%), FluA (9.03% vs. 4.00%), and FluB (3.39% vs. 1.22%) compared with inpatients (all *P* < 0.001). Conversely, inpatients had higher rates of RV (18.71% vs. 14.97%) and RSV (10.92% vs. 7.64%) (both *P* < 0.001). MP detection rates did not differ between outpatients and inpatients (14.12% vs. 13.53%, *P* = 0.091). Clinical setting-related differences are shown in Fig. 1B and detailed in Supplementary Table S2.

**Table 2:** Pathogen composition differences by visit type (Outpatient vs. inpatient)

Variables	Total (n = 68982)	Inpatient (n = 56963)	Outpatient (n = 12019)	Statistic	P
FluB, n(%)	1103 (1.60)	696 (1.22)	407 (3.39)	χ^2^=295.52	<.001
HRV, n(%)	12454 (18.05)	10655 (18.71)	1799 (14.97)	χ^2^=93.69	<.001
RSV, n(%)	7139 (10.35)	6221 (10.92)	918 (7.64)	χ^2^=115.31	<.001
FluA, n(%)	3363 (4.88)	2278 (4.00)	1085 (9.03)	χ^2^=541.10	<.001
MP, n(%)	9668 (14.02)	8042 (14.12)	1626 (13.53)	χ^2^=2.86	0.091
AdV, n(%)	8332 (12.08)	5425 (9.52)	2907 (24.19)	χ^2^=2009.38	<.001

χ^2^: Chi-square test

### Age-specific pathogen distribution

Age-stratified analysis revealed distinct pathogen patterns across age groups (Supplementary Table S3; Fig. 1C; all *P* < 0.001). RSV prevalence declined progressively with age, from 16.22% in infants to 2.60% in adolescents. In contrast, MP prevalence increased with age, peaking at 29.91% in school-aged children before declining to 13.24% in adolescents. RV remained common across all age groups, with the highest prevalence in toddlers (21.40%). AdV peaked in preschoolers (17.07%) and school-aged children (16.47%). FluA and FluB prevalence increased with age, reaching their highest levels in school-aged children (6.17% and 3.23%, respectively).

### Seasonal variation of pathogens

As illustrated in Fig. 1D, the seasonal dynamics of AdV and MP exhibited distinct peaks, while other pathogens showed more predictable winter-predominant patterns. Detailed comparative statistics for these seasonal variations are provided in Supplementary Table S4. RSV peaked in winter (18.34%), with minimal detection in summer (1.93%). FluA and FluB also peaked in winter (9.27% and 4.36%, respectively), with FluB nearly absent in summer (0.04%). RV peaked in autumn (23.63%) and remained elevated in spring (20.44%), with lower rates in summer (16.26%) and winter (12.93%). MP showed the highest detection in autumn (18.37%), followed by summer (13.94%) and winter (14.20%). AdV peaked in summer (17.88%), with moderate rates in autumn (13.00%) and winter (9.51%). All these seasonal differences were statistically significant (all *P* < 0.001; Supplementary Table S4; Fig. 1D).

### Post-pandemic temporal dynamics

**Fig. 2:**
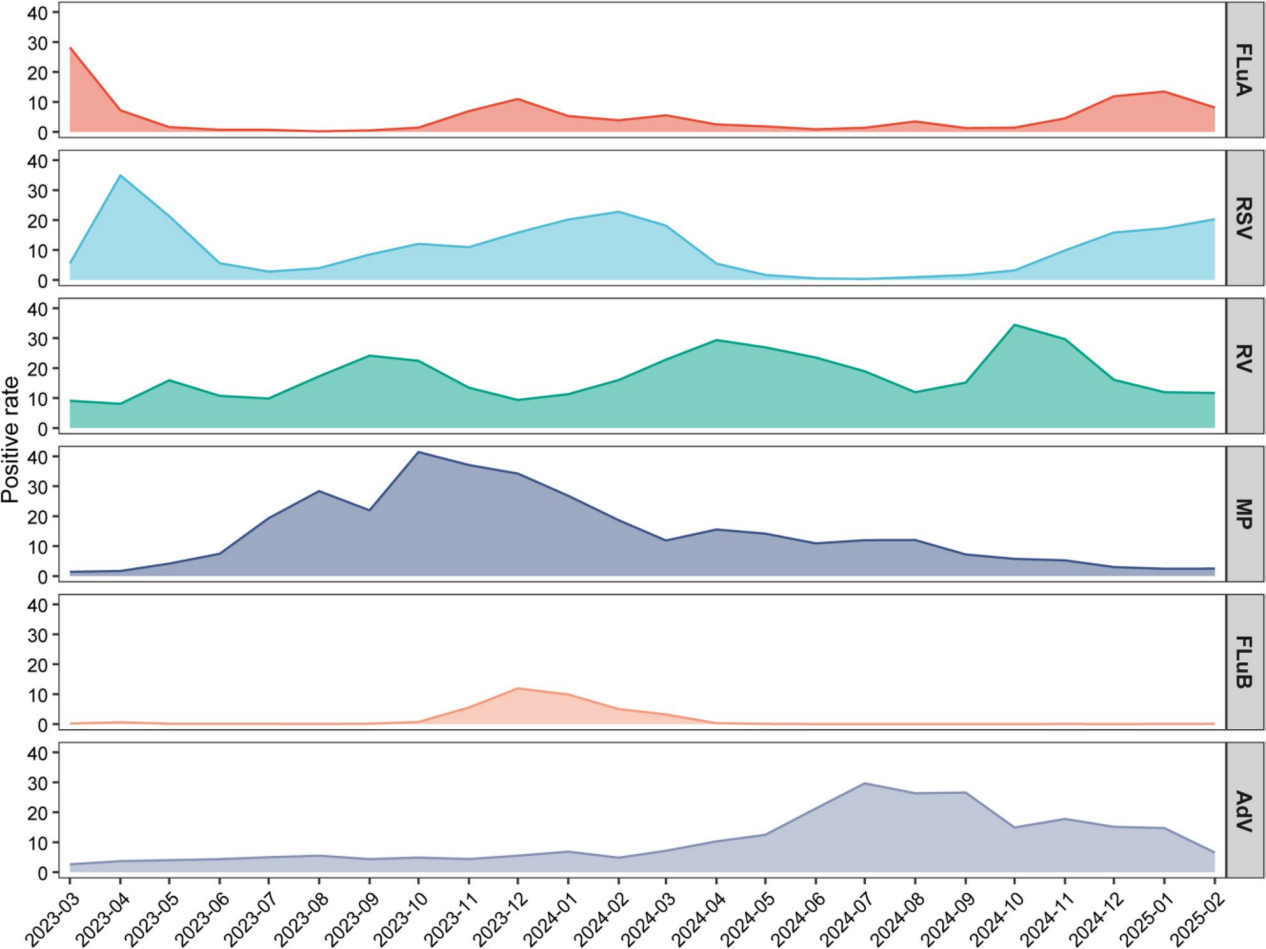
Temporal trends in the positive rates of respiratory pathogens from March 2023 to February 2025

As shown in Fig. 2, a distinct sequential pattern of pathogen detection was observed following the relaxation of COVID-19 restrictions. Among all ARI cases tested, FluA positivity surged to 28.21% in March, followed by an RSV peak of 34.92% in April. RV reached 15.94% in May and remained elevated through autumn, peaking at 34.45% in October. MP activity increased gradually, peaking at 41.41% in October. AdV rose from June onward, peaking at 29.66% in July and remaining elevated through the end of the year. Figure 2 clearly illustrates the post-pandemic resurgence patterns of these respiratory pathogens.

### Co-infection patterns

**Fig. 3:**
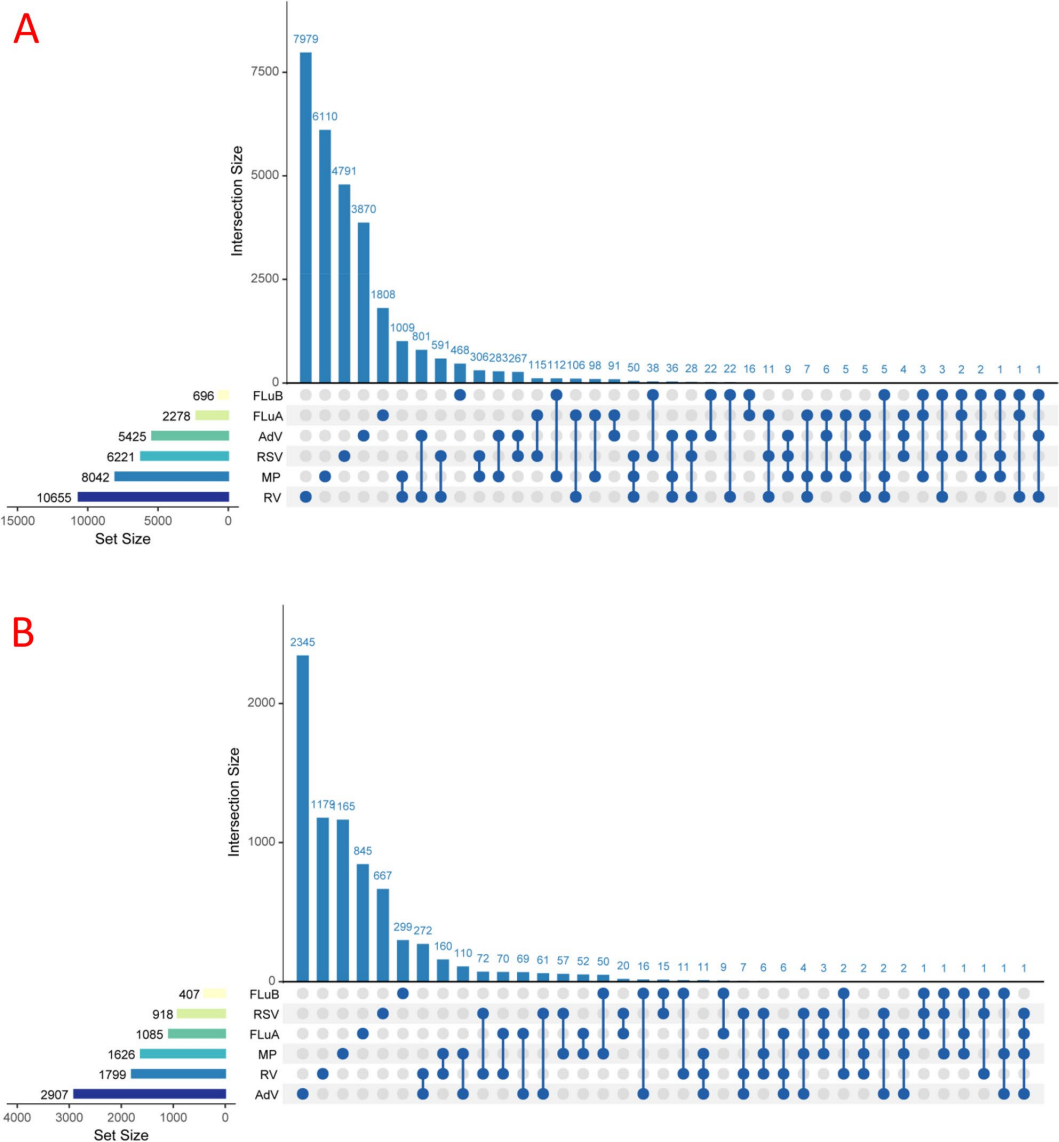
UpSet plots of co-infection patterns in inpatients (**A**) and outpatients (**B**). The left horizontal bar plot shows the total number of positive cases for each pathogen (Set Size). The vertical bar plot shows the number of cases for each pathogen combination (Intersection Size). The matrix below indicates which pathogens are present in each combination

**Fig. 4:**
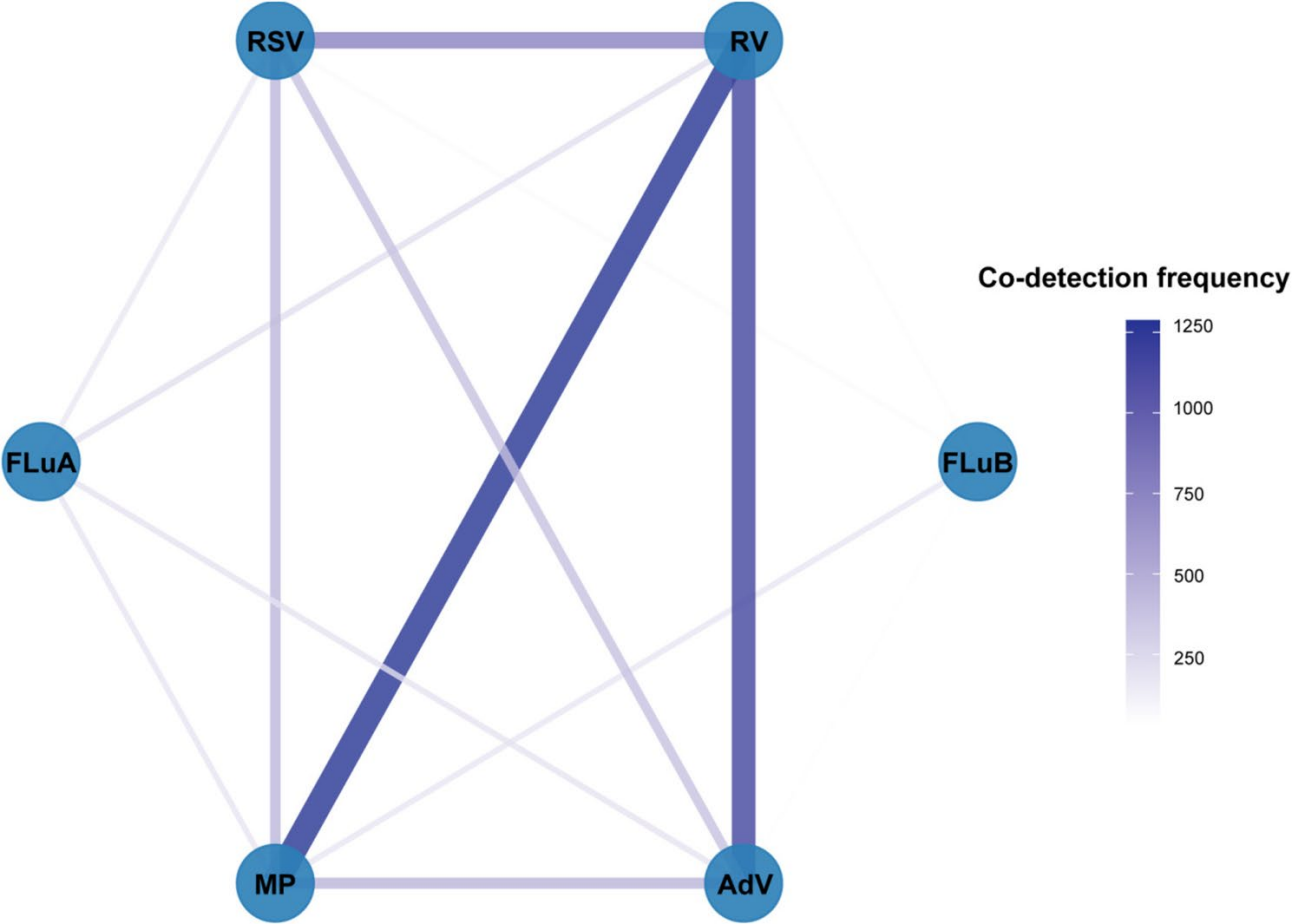
Circular co-infection network of the six respiratory pathogens. Edge width and color intensity represent the co-detection frequency between two pathogens (darker/thicker = higher frequency)

Co-infection frequencies were higher in inpatients than in outpatients, as shown in the UpSet plots (Fig. 3A: inpatients; Fig. 3B: outpatients). Among inpatients, the most common co-infection pair was RV–MP (*n* = 1,009), followed by AdV–RV (*n* = 801) and MP–RSV (*n* = 591). Among outpatients, AdV–RV was the most frequent (*n* = 272), followed by RV–MP (*n* = 160) and RV–FluA (*n* = 110). The circular network analysis (Fig. 4) showed that RV had the highest node degree, with the strongest edge connecting RV and MP, followed by RV–AdV and MP–RSV. Weaker associations were observed for FluA–MP and RV–FluA.

### Multivariable analysis

**Fig. 5:**
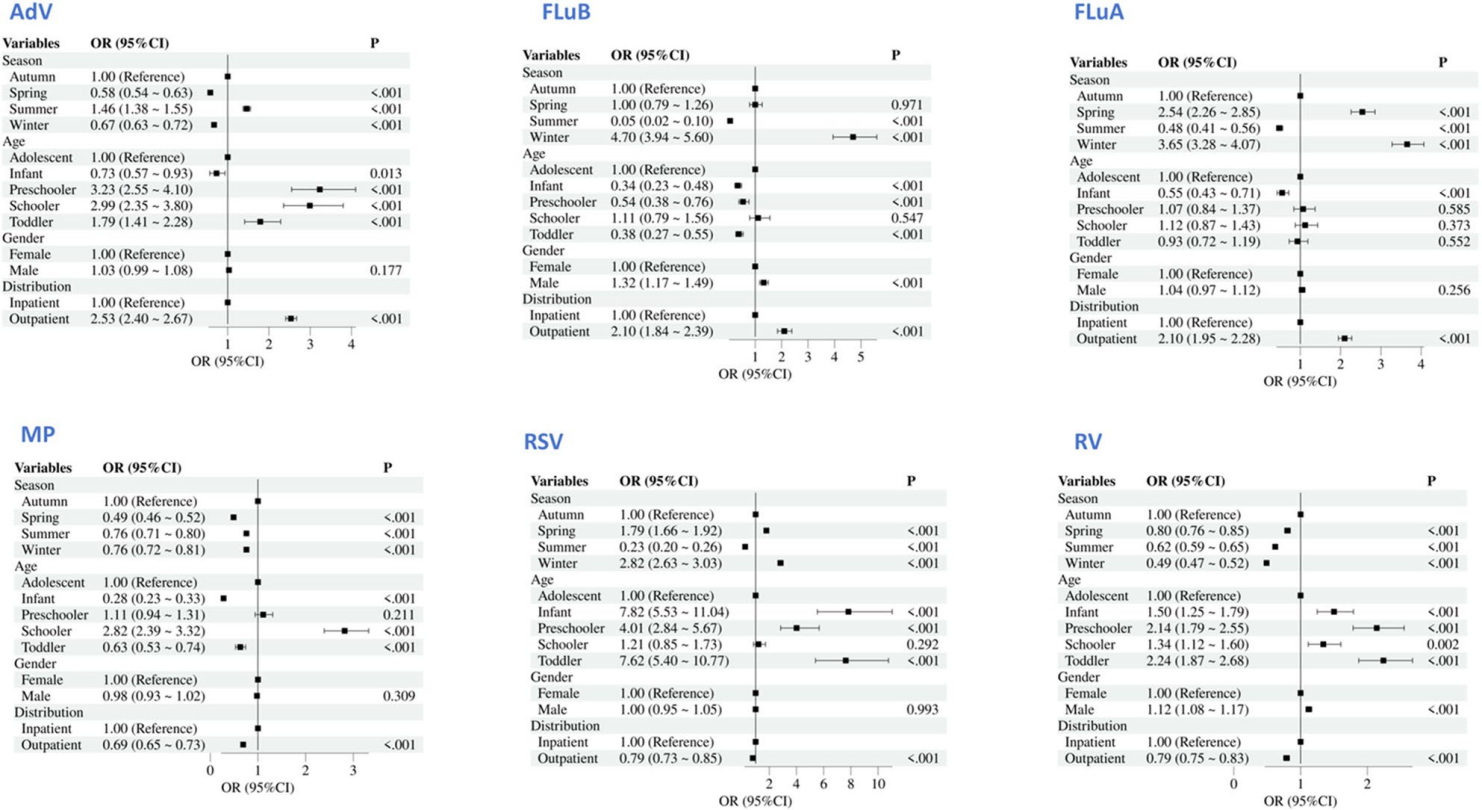
Multivariable logistic regression analysis of factors associated with detection of six respiratory pathogens. This figure shows the results of logistic regression analysis, with the x-axis representing the odds ratio (OR), and the y-axis listing the variables tested. Data are presented for different seasons (autumn, spring, summer, and winter) and age groups (infant, preschooler, schooler, toddler, adolescent). Each pathogen is analyzed separately, with significant p-values (< 0.001) indicated for various comparisons

Multivariable logistic regression (Fig. 5) identified age, season, and clinical setting as independent predictors of pathogen detection. Using adolescents as the reference for age, infants had higher odds of RSV (OR = 7.82; 95% CI: 5.53–11.04), and school-aged children had higher odds of MP (OR = 2.82; 95% CI: 2.39–3.32). Regarding season (reference: autumn), winter was associated with higher odds of RSV (OR = 2.82; 95% CI: 2.63–3.03) and Flu B (OR = 4.70; 95% CI: 3.94–5.60), while summer was associated with higher odds of AdV (OR = 1.46; 95% CI: 1.38–1.55). Finally, using inpatient status as the reference, outpatient status was associated with higher odds of AdV (OR = 2.53; 95% CI: 2.45–2.62) but lower odds of RSV (OR = 0.79; 95% CI: 0.75–0.83).

## Discussion

### Epidemiological landscape and the post-pandemic resurgence

Our study, involving 68,982 pediatric patients, provides a robust, longitudinal assessment of the respiratory pathogen landscape in the post-pandemic era. The overall positivity rate of 53.1% and the distinct age-dependent shift—from 38.8% in infants to 63.5% in school-age children—reflect a marked shift in pediatric immunity with age, likely shaped by cumulative exposure and natural immune maturation. The ‘immunity debt’ hypothesis, initially proposed to explain the resurgence of non-SARS-CoV-2 pathogens after the lifting of non-pharmaceutical interventions (NPIs), is clearly manifested here as a staggered, pathogen-specific rebound [8]. While the ‘immunity debt’ hypothesis provides a compelling framework, it remains a subject of ongoing debate. Other mechanisms, such as viral interference (where the infection of one virus may inhibit the transmission of another), likely also play a crucial role in the observed pathogen-specific rebound [9].

Unlike the synchronized seasonal surges seen in pre-pandemic years, our data reveal a sequential pattern: Influenza A and RSV dominated the early-year recovery, whereas MP and AdV exhibited a delayed, “slow-burn” resurgence [10, 11]. This sequential rebound might be explained by the varying lengths of duration for naturally acquired immunity across different pathogens. While influenza and RSV, characterized by rapid transmission and shorter immunity duration, bounced back immediately after the relaxation of NPIs, pathogens with longer incubation periods and more complex transmission dynamics, such as MP, required a longer “incubation phase” to regain their endemic equilibrium [11].

### Pathogen-specific dynamics and synergistic interactions

Our findings highlight the profound impact of RSV on pediatric hospitalization, aligning with global surveillance data [12, 13]. Given the winter peak and high positivity and hospitalization rates in infants, our study provides local epidemiological data that could inform the timing of RSV preventive measures, such as maternal immunization or monoclonal antibodies. In contrast, influenza was more frequently detected in outpatients, particularly among school-aged children, confirming their role as primary community spreaders. Therefore, promoting influenza vaccination in schools may not only reduce community viral load but also protect vulnerable infants through herd immunity.

RV and AdV: The higher detection rate of RV in hospitalized patients compared with outpatients (18.71% vs. 14.97%) suggests that RV may contribute to more severe respiratory illness. This is particularly concerning for children with underlying conditions, such as asthma or immunodeficiency, which are known risk factors for severe viral lower respiratory tract infections [14, 15]. Given the lack of specific antivirals or vaccines for RV, during periods of increased RV activity (especially in autumn and spring), strengthening non-pharmacological interventions—such as enhancing hand hygiene, mask-wearing, and improving ventilation in childcare facilities and schools—will help reduce transmission. Conversely, the summer AdV peak (17.88%) presents a diagnostic blind spot. AdV infection can cause persistent high fever and significantly elevated inflammatory markers, often leading to misdiagnosis as bacterial infections and subsequent empirical antibiotic use, which further delays the identification of AdV-associated respiratory infections. Our data suggest that AdV is a significant seasonal contributor to pediatric respiratory morbidity during summer. Implementing targeted molecular diagnostics can optimize clinical management and promote rational antibiotic use, although its direct impact on antibiotic utilization requires further prospective validation [16, 17]. High-risk children for severe AdV infection include those who are immunocompromised, have underlying cardiopulmonary disease, or are < 2 years of age [18]. For these patients, early detection of AdV is particularly important as it may enable consideration of antiviral therapy (e.g., cidofovir) in severe cases, although its use remains off-label and requires careful monitoring of renal function [19].

MP and Polymicrobial Challenges: The autumn peak of MP is a defining feature of our findings, with the highest detection rate in school-aged children (29.91%). The delayed resurgence of MP (peaking in late 2023) is consistent with its longer incubation period and slower, cyclical transmission dynamics [20]. Notably, MP detection rates did not differ between outpatients and inpatients (14.12% vs. 13.53%, *P* = 0.091), suggesting that MP causes substantial morbidity across both settings. A critical observation in our study is the high correlation between MP and RV co-infections. We propose that a potential synergistic mechanism may exist, wherein MP-induced epithelial damage might compromise mechanical barriers, thereby facilitating viral co-infection. However, this mechanism remains speculative and requires further mechanistic investigation [21, 22]. This polymicrobial synergy is increasingly recognized as a significant predictor of treatment failure in pediatric pneumonia, highlighting the urgency of shifting toward a multi-pathogen management strategy [9].

### Diagnostic stewardship and clinical management

Although multiplex PCR is a powerful tool, its high cost limits routine use in all patients. Our data show that pathogen positivity rates vary significantly by season and age (winter: RSV/influenza dominant; summer: AdV peak; autumn: MP peak), making a “one-size-fits-all” testing strategy inefficient and uneconomical. A season- and age-stratified “risk-based” testing approach (prioritizing influenza/RSV in winter, adding AdV in summer, and focusing on MP in autumn) can improve diagnostic yield without substantially increasing costs, thus being more clinically feasible. For hospitalized children, the identification of co-infections (e.g., RV-MP or RV-AdV) should serve as a signal for enhanced clinical monitoring and supportive care, as these patients are at a higher risk of prolonged morbidity and may require more intensive management, despite limited specific antivirals for some pathogens.

### Strengths and limitations

The primary strength of this study is the large-scale, longitudinal dataset, which offers a comprehensive “snapshot” of the post-pandemic respiratory landscape. However, several limitations must be acknowledged. First, the absence of granular clinical severity metrics (e.g., ICU admission duration, oxygen requirement) prevents a definitive correlation between pathogen presence and specific clinical outcomes. Second, the use of a binary Ct threshold (< 40) does not fully account for viral load dynamics, making it difficult to distinguish between active infection and prolonged viral shedding that may not require clinical intervention. Third, the restriction to a six-pathogen panel potentially under-represents the contribution of other significant respiratory viruses (e.g., HMPV or SARS-CoV-2 variants). Finally, as a single-center study in Chengdu, our findings may be influenced by local environmental and demographic factors; future multicenter studies across different geographic regions are warranted to validate these findings and further refine risk-stratified diagnostic algorithms.

## Conclusion

This study provides a systematic description of age and seasonal patterns of six respiratory pathogens in post-pandemic Chengdu. Key findings: RSV peaks in winter (infants), MP peaks in autumn (school-aged children), AdV peaks in summer, and RV is highly prevalent with frequent MP/AdV co-infections in hospitalized children. Our findings suggest that a season- and age-stratified approach to clinical monitoring—such as prioritizing influenza/RSV surveillance in winter and focusing on MP in autumn—may help optimize diagnostic and therapeutic resource allocation in pediatric settings.

## Supplementary Information

Supplementary Material 1.

Supplementary Material 2.

Supplementary Material 3.

## Data Availability

The datasets generated and analyzed during the current study are not publicly available due to patient privacy and institutional ethical restrictions but are available from the corresponding author (Yinghong Fan) on reasonable request, subject to approval by the Ethics Committee of Chengdu Women’s and Children’s Central Hospital.

## Abbreviations

AdV: Adenovirus
ARI: Acute respiratory infection
CI: Confidence interval
Ct: Cycle threshold
FluA: Influenza A virus
FluB: Influenza B virus
IRB: Institutional Review Board
MP: *Mycoplasma pneumoniae*
NPI: Non-pharmaceutical intervention
OR: Odds ratio
PCR: Polymerase chain reaction
RSV: Respiratory syncytial virus
RV: Rhinovirus
