# Supplementary material for: Epidemiological characteristics and seasonal dynamics of six respiratory pathogens in children: a 2-year retrospective cross-sectional study in Chengdu, China
Source: BMC Pediatr. 2026 Jun 3;26:718. doi: 10.1186/s12887-026-07063-3 (PMC13449576; doi:10.1186/s12887-026-07063-3)
Supplement: Supplementary file 1 [file 12887_2026_7063_MOESM1_ESM.pdf]

伦理审查批件

批件号：科研伦审 2025（77）号

项　目<br>名　称	儿童呼吸道病原核酸检测回顾性研究：流行病学特点与相关影响因素分析		
类　别	1.医学科研（√）2.新技术新业务（　）3.辅助生殖（　）4.产前诊断（　）		
审　查<br>方　式	1.会议审查（ ）2.快速审查（√）		
主 审<br>委 员	喻琳麟、刘青松		
项目负<br>责任人	陈洪佳　主治医师	所　属<br>部　门	儿童呼吸内科
审查文件	**初审文件**<br>1.伦理审查申请表<br>2.临床研究方案（版本号：V1.0，版本日期：2025 年 02 月 14 日）<br>3.免除知情同意或免除知情同意部分要素申请表<br>4.主要研究者简历<br>5.研究小组成员名单<br>6.IIT 临床研究科学性审查意见表		
根据我国相关法律、法规和规范，　以及伦理原则，经本伦理委员会审查决定	同意<br>研究负责人必须严格使用经审查同意的知情同意书文本和研究方案。如伦理审查批件失效时不能完成所有的临床研究（包括统计分析），请在本批件失效前一个月，递交持续审查申请。<br>1.如研究结束并在审查有效期内，请递交结题报告；<br>2.研究中发生涉及受试者或其他人风险的任何 SAE 或非预期的不良事件，应立刻报告本伦理委员会；<br>3.任何研究方案、知情同意书的修改包括研究人员的变更，必须递交研究方案修改申请表，经伦理委员会审查获得批准后执行；<br>4.出现没有遵从方案开展研究的情况，或可能对受试者的权益/健康、以及研究的科学性造成不良影响等情况，请及时提交违背方案报告至本伦理委员会；<br>5.请按照跟踪审查的频率要求，在预计审查日期前 1 月提交跟踪审查报告；<br>6.申请人暂停或提前终止临床试验，请及时提交暂停/终止研究报告；<br>7.如涉及人类遗传资源审批，需取得中国人类遗传资源管理办公室出具的相关批件后，方可开展临床研究。		
伦理审查批件<br>有效期	2025 年 6 月 17 日 至<br>2026 年 6 月 16 日	跟踪审查<br>频率	12 个月
主任委员签字		日期	2025 年 6 月 17 日

声明：本伦理委员会的职责、人员组成、运行和记录遵循中华人民共和国原卫生和计划生育委员会2016年颁布的《涉及人的生物医学研究伦理审查办法》和《赫尔辛基宣言》的伦理原则，并遵守中国的相关法律及法规。		
联系地址：成都市青羊区日月大道1617号	联系电话：028-61866015；028-61866009	邮箱：lunliwyh@163.com
