# Supplementary material for: Epidemiological characteristics and seasonal dynamics of six respiratory pathogens in children: a 2-year retrospective cross-sectional study in Chengdu, China
Source: BMC Pediatr. 2026 Jun 3;26:718. doi: 10.1186/s12887-026-07063-3 (PMC13449576; doi:10.1186/s12887-026-07063-3)
Supplement: Supplementary file 2 [file 12887_2026_7063_MOESM2_ESM.pdf]

申报编号：23NSFSC5420　　　　立项编号：2023NSFSC1469

# 四川省自然科学基金项目任务书
# （青年基金项目）

**项目名称：** MiRNAs在儿童肺炎支原体肺炎中的表达及对疾病炎性细胞因子调控的作用研究

**承担单位：** 成都市妇女儿童中心医院　（盖章）

**项目负责人：**　　　　（签字）

**推荐单位：** 成都市科学技术局

**立项经费：** 10　（万元）

**项目起止年限：** 2023-01-01 至 2024-12-31

四川省科技计划项目任务书正式版

## 四川省科学技术厅制

扫描全能王
3亿人都在用的扫描App

# 九、任务书签订各方盖章及意见

| | | | |
|---|---|---|---|
| 甲方 | 单位名称 | 四川省科学技术厅 | （项目合同章） |
| | 分管厅领导 | （签章） | |
| | 分管处室负责人 | （签章） | |
| | 项目管理人 | 喻茹 | |
| | 电话及传真 | 86729286 | 年 月 日 |
| 乙方 | 牵头单位名称 | 成都市妇女儿童中心医院 | （牵头单位公章） |
| | 地址及邮编 | 成都市青羊区日月大道1617号，610091 | |
| | 电话及传真 | (028)61866000-6682 | |
| | 开户银行 | 中信银行东城根街支行 | |
| | 帐 号 | 7411510183100000168 | 2023年4月12日 |
| 丙方 | 推荐单位名称 | 成都市科学技术局 | （单位公章） |
| | 电话及传真 | | 2023年4月12日 |

四川省科技计划项目任务书正本

扫描全能王
3亿人都在用的扫描App
