# Supplementary material for: Epidemiological characteristics and seasonal dynamics of six respiratory pathogens in children: a 2-year retrospective cross-sectional study in Chengdu, China
Source: BMC Pediatr. 2026 Jun 3;26:718. doi: 10.1186/s12887-026-07063-3 (PMC13449576; doi:10.1186/s12887-026-07063-3)
Supplement: Supplementary file 3 [file 12887_2026_7063_MOESM3_ESM.pdf]

Ethics Approval Document (English Translation)
Ethics Review Approval
Approval No.: Scientific Research Ethics Approval No. 2025 (77)

Project Title:

Quality Control Study of Nucleic Acid Testing for Respiratory Pathogens in Children: Analysis of Epidemiological Characteristics and Related Influencing Factors

Category:

☑ Medical Scientific Research

☐ New Technology / New Clinical Practice

☐ Assisted Reproduction

☐ Prenatal Diagnosis

Review Type:

☐ Full Board Review

☑ Expedited Review

Review Committee Members:

Ha Linxin, Liu Qingbo

Principal Investigator:

Chen Hongjia, Chief Physician

Department:

Department of Pediatric Respiratory Medicine

Submitted Documents Reviewed:
Ethics Review Application Form
Clinical Research Protocol (Version: V1.0; Date: February 14, 2025)
Application Form for Waiver of Informed Consent and Key Elements of Waiver
Curriculum Vitae of the Principal Investigator
List of Research Team Members
IIT Clinical Research Scientific Review Opinion Form

Approval Decision:
Approved

Approval Conditions:
The principal investigator must strictly conduct the research in accordance with the approved informed consent documents and research protocol. If the ethics approval expires before completion of all clinical research activities (including statistical analysis), an application for continuing review must be submitted within one month prior to expiration.

If the study is completed within the validity period of the ethics approval, a study completion report must be submitted.
Any serious adverse events (SAEs) or unanticipated problems involving risks to participants or others that occur during the study must be reported promptly to the Ethics Committee.
Any modifications to the research protocol, informed consent documents, or changes in research personnel must be submitted as amendments and may be implemented only after approval by the Ethics Committee.
If any situation arises that prevents the study from being conducted as planned or significantly affects participants' rights, safety, or the scientific integrity of the study, a written report must be submitted promptly to the Ethics Committee.
Progress reports must be submitted one month prior to the scheduled continuing review, in accordance with the required review frequency.
If the investigator proposes early termination of the clinical study, a termination report must be submitted.
For studies involving human genetic resources, approval must be obtained from the China Administration of Human Genetic Resources before commencing the clinical study.
Validity Period of Ethics Approval:
From June 17, 2025 to the date of continuing review

Continuing Review Frequency:

Every 12 months

Chairperson of the Ethics Committee
Date:
June 17, 2025

Statement

This Ethics Committee performs its duties in accordance with the Measures for the Ethical Review of Biomedical Research Involving Human Subjects issued by the National Health and Family Planning Commission of the People’s Republic of China in 2016, the Declaration of Helsinki, and relevant Chinese laws and regulations.

Contact Information:

Address: No. 161, Riyue Avenue, Qingyang District, Chengdu, China

Telephone: +86-28-61866015

Fax: +86-28-61866009

Email: lunliwyh@163.com
